# A Fast Response Ammonia Sensor Based on Coaxial PPy–PAN Nanofiber Yarn

**DOI:** 10.3390/nano6070121

**Published:** 2016-06-23

**Authors:** Penghong Liu, Shaohua Wu, Yue Zhang, Hongnan Zhang, Xiaohong Qin

**Affiliations:** Key Laboratory of Textile Science & Technology, Ministry of Education, College of Textiles, Donghua University, No. 2999 North Renmin Road, Songjiang District, Shanghai 201620, China; lph_1989@sina.com (P.L.); wushaohua87@163.com (S.W.); zhangyue1989@163.com (Y.Z.)

**Keywords:** electrospinning, polypyrrole, coaxial polypyrrole (PPy)–polyacrylonitrile (PAN) nanofiber yarn, ammonia sensor

## Abstract

Highly orientated polypyrrole (PPy)–coated polyacrylonitrile (PAN) (PPy–PAN) nanofiber yarn was prepared with an electrospinning technique and *in-situ* chemical polymerization. The morphology and chemical structure of PPy–PAN nanofiber yarn was characterized by scanning electron microscopy (SEM), field emission scanning electron microscopy (FESEM), transmission electron microscopy (TEM), and fourier transform infrared spectroscopy (FTIR), which indicated that the PPy as the shell layer was homogeneously and uniformly polymerized on the surface of PAN nanofiber. The effects of different concentration of doping acid on the responses of PPy–PAN nanofiber yarn sensor were investigated. The electrical responses of the gas sensor based on the PPy–PAN nanofiber yarn to ammonia were investigated at room temperature. The nanoyarn sensor composed of uniaxially aligned PPy–PAN nanofibers with a one-dimensional structure exhibited a transient response, and the response time was less than 1 s. The excellent sensing properties mentioned above give rise to good potential application prospects in the field of ammonia sensor.

## 1. Introduction

Recently, gas sensors have been developed very fast all over the world due to their wide application in various fields such as environmental monitoring, modern industry and agriculture, military affairs, national defense, and even disease diagnosis [1,2,3,4,5,6,7,8,9]. A wide range of materials, such as metal oxides and conducting polymer, are served as sensing elements [10,11,12,13]. It is well known that metal oxide sensor is operated at high temperatures. Whereas the conducting polymers sensors have the potential of working near room temperature [14]. Conducting polymers like polypyrrole (PPy), polyaniline (PANI), and polythiophene contain π-electron-conjugated carbon chains [15,16], which have many excellent properties such as electrochemical reversibility, relatively environmental stability, controllable electrical conductivity, good mechanical performance, and ease of preparation using chemical and electrochemical synthesis methods. PPy is a p-type semiconductor, which have sensitive properties to many gases such as ammonia, ethanol, methanol, acetone, HCl, and volatile organic compounds [17,18,19,20,21,22,23].

There are plenty reports on the ammonia gas sensor using nanosized PPy as sensing materials such as PPy thin film, PPy wires, and nanocomposites of PPy with other polymers or metal oxides [24,25,26,27]. Nanomaterials with high surface-to-volume ratio will increase sensing activities. However, most of these nanosized materials are usually prepared in a randomly oriented manner. This randomly oriented structure may limit its application in the field of electronic, photonic and sensors. The nanofiber microyarns [28,29,30,31,32] are composed of one-dimensionally aligned nanofibers. These assembled forms are beneficial in providing an effective means of controlling the resistance of sensors and modulating the direction of charge transfers [33].

In this paper, we prepared a polypyrrole (PPy)-coated polyacrylonitrile (PAN) (PPy–PAN) nanofiber yarn ammonia gas sensor with transient response characteristics. First, polyacrylonitrile (PAN) nanofiber yarns were electrospun by a novel and modified electrospinning nano-yarn machine [28] (designed by our own research group). Then, the PPy nanostructure layer was coated by simple and straightforward *in-situ* chemical polymerization on the surface of PAN yarns. PPy–PAN sensor with high sensitivity and transient response time in an ammonia atmosphere was demonstrated. The concentration of doping acid was able to affect sensing properties of the PPy–PAN sensor. We conclude that PPy–PAN nanofiber yarns as a flexible electronic material have potential application in the intelligent electronics field.

## 2. Results

### 2.1. Characterization of the Coaxial PPy–PAN Nanofiber Yarns

**Figure 1:**
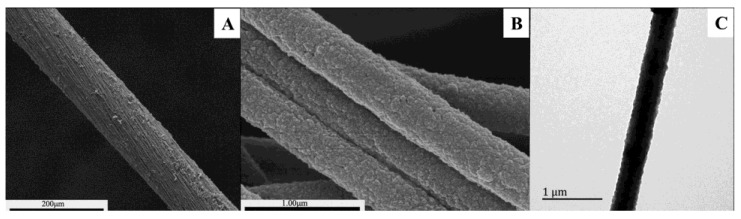
(**A**) Scanning electron microscopy (SEM) image of coaxial polypyrrole (PPy)-coated polyacrylonitrile (PAN) nanofiber yarn; (**B**) Field emission scanning electron microscopy (FESEM) image of aligned PPy–PAN nanofibers; (**C**) Transmission electron microscopy (TEM) image of PPy–PAN nanofiber.

The surface morphology and size of the coaxial PPy–PAN nanofiber yarns could be observed by the SEM and TEM images shown in Figure 1. The images of Figure 1A,B shows that the PPy–PAN nanofiber yarns were composed of orientated and uniform PPy-coated PAN nanofibers. The average diameter of the nanofiber yarns was 105 μm, and the inner nanofibers had a diameter of 406 ± 34 nm. It can be seen that the structure of the yarns was tightly assembled with orientated alignment nanofibers. The degree of twist in the yarns can also be detected from Figure 1A. It can be observed that the pyrrole was polymerized on the surface of the inner PAN nanofibers. The PPy layer was smooth and uniform. The polymerization of pyrrole on the surface of the nanofibers did not transform and destroyed the structure and morphology of PAN nanofiber yarns, which was only a matter of adding the diameter of yarns. The formation of PPy on the surface of the nanofibers can be detected with the naked eye through the change in yarn color. The color of as-prepared electrospinning PAN nanofiber yarn was white. When it was dipped into the reaction solution, the existence of FeCl_3_ changed the color of the yarns from white to yellowish. The surface of PAN nanofibers was coated with a PPy layer due to the chemical redox process of pyrrole and FeCl_3_, which ultimately resulted in the formation of black yarns. The detailed morphology and structure of the PPy film can be characterized and investigated by the TEM (Figure 1C). The PPy–PAN nanofiber had a core-shell structure with a uniform and homogeneous conducting PPy layer. PPy particles that were homogeneous coated on the surface of the nanofiber had no obvious agglomeration. The noises from the structural defects can be suppressed by the homogeneous and uniform PPy layer [34]. The PPy-coated on the surface of PAN nanofibers with a high specific surface area can provide more active sites of PPy for gas adsorption [35]. The straight yarn structure and inner uniaxially aligned PPy–PAN nanofibers with a one-dimensional structure that has a longer conjugation length will accelerate the charge transport [33,36], which makes it potential material of various sensor applications.

**Figure 2:**
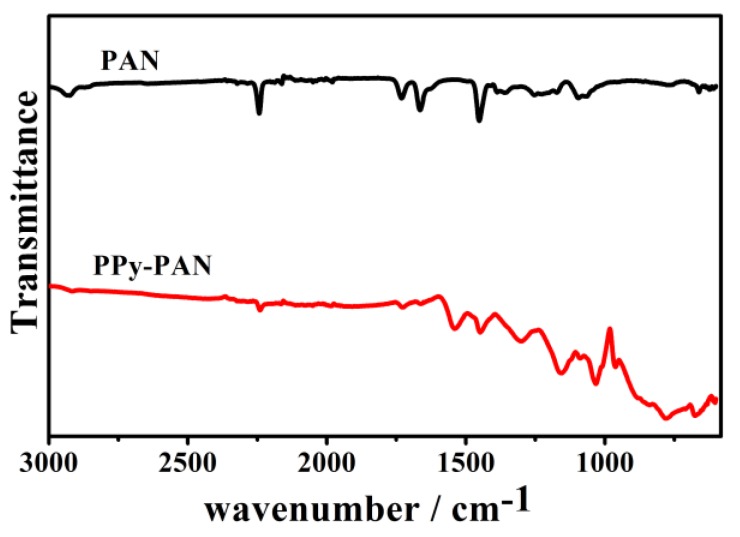
Fourier transform infrared spectroscopy (FTIR) spectra of PAN and PPy–PAN coaxial nanofiber yarn.

The successful coating of PPy on the PAN nanofibers was further certified by FTIR. Figure 2 indicates the spectral features of the PAN and PPy polymer. The peak at 2925 cm^−1^ was assigned to the methylene (–CH_2_–) stretching vibration that belonged to the pure PAN nanofiber. The characteristic peaks at 2243 and 1454 cm^−1^ corresponded to the stretching vibration of the nitrile groups (–CN–) and the bending vibration of the methylene of the PAN, respectively. [37,38]. The characteristic peaks of PPy–PAN nanofiber yarns at 2925 and 2243 cm^−1^ were significantly reduced, which indicated that the synthesis of polypyrrole weakened the original characteristic functional group in PAN. The bands at 1541 and 1448 cm^−1^ were assigned to the presence of five-membered PPy ring-stretching and conjugated C–N stretching vibration bands, respectively. [39,40]. The peak around 1298 and 1154 cm^−1^ represented C–N stretching and the C–H deformation vibration. The peaks at 960 and 775 cm^−1^ were attributed to C–H in- and out-plane deformation. The peak around 1032 cm^−1^ was the characteristic absorption peak of SO_3_^−^, which indicated that the phenyl ring was substituted by a sulfonic acid group. The peak commonly at 1250 cm^−1^ represented the C–H and C–N in-plane deformation. Infrared spectroscopy (IR) band shifting to 1298 cm^−1^ was attributed to Cl^−^ doped PPy.

### 2.2. NH_3_ Sensing

**Figure 3:**
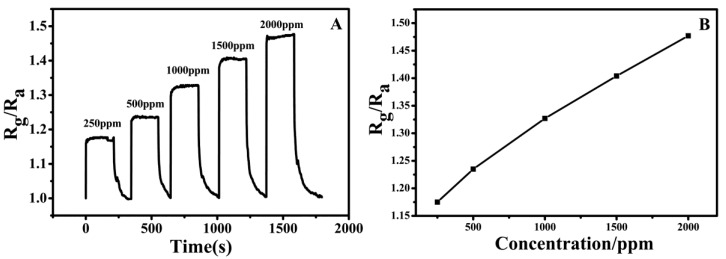
(**A**) Response of PPy–PAN nanoyarn exposed to different ammonia concentrations; (**B**) The linear relationship between the sensitivity of the PPy–PAN nanoyarn and different ammonia concentrations. (The concentration of *p*-toluenesulfonic acid (*p*-TSA) was 0.004 M).

**Figure 4:**
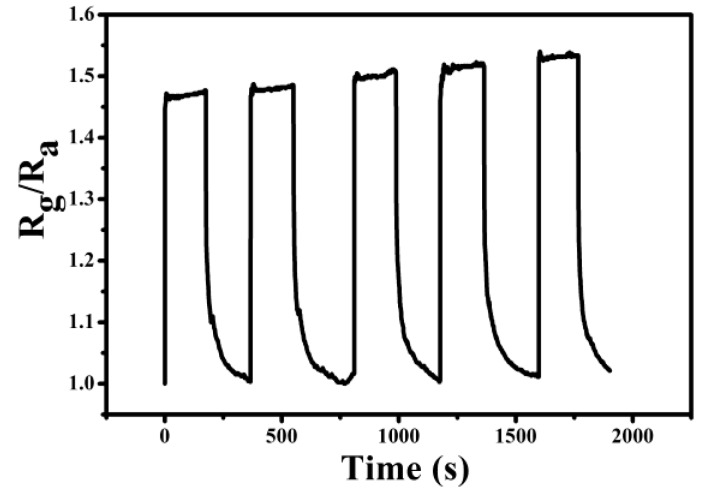
The sensitivity of the PPy–PAN nanoyarn exposed to an ammonia concentration of 2000 ppm during cycle tests. (The concentration of *p*-toluenesulfonic acid (*p*-TSA) was 0.004 M).

The ammonia sensing experiment of the PPy–PAN coaxial nanofiber yarn was operated at room temperature, and the result is presented in Figure 3. The sample was the PPy–PAN nanofiber yarn sensor protonated with 0.004 M *p*-toluenesulfonic acid (*p*-TSA). The sensor was exposed to different concentrations of ammonia, and the gas sensitivity curves of PPy–PAN yarn were recorded in Figure 3A. The sensitivity (S) of sensor is defined as S = R_g_/R_a_, where R_g_ is the resistance of the sensor in ammonia gas, and R_a_ is the sensor in air. It was seen that the resistance of the sensor increased along with the afflux of ammonia. When the gas was pumped out, the resistance decreased rapidly at the beginning and then slowly reached the initial resistance ultimately. From the curve of Figure 3A, the response time (defined as the time to achieve a 90% total resistance change) of the sample was less than 1 s, which can be seen as the transient response. However, the recovery time was around 60 s. The transient response time of the sensor may be attributed to the synergistic combination of advantages of PPy and the structure of uniaxially aligned PPy-coated PAN nanofiber yarn, which is a major breakthrough. As shown in Figure 3B, the sensitivity increased linearly with a rising concentration of ammonia. The reproducibility of the PPy–PAN nanofiber yarn gas sensor during the cycle test was also investigated, and results are illustrated in Figure 4. It can be seen that the sensitivity of the nanoyarn exposed in 2000 ppm ammonia vapor were highly reproducible during the tests of the five cycles. The sensitivity was consistent during every cycle test.

**Figure 5:**
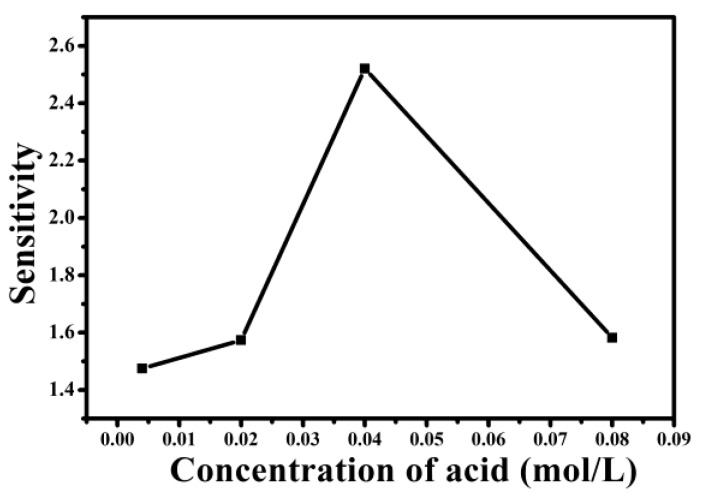
Sensitivity of the PPy–PAN nanoyarn with different *p*-TSA concentrations exposed to an ammonia concentration of 2000 ppm.

Apart from the morphology and structure of nanofiber yarn, the polymerization conditions of pyrrole might also have an effect on the sensitivity of the sensor. The different concentrations of doping acid (*p*-TSA, 0.004 M, 0.02 M, 0.04 M, and 0.08 M) were prepared. Figure 5 shows that the sensitivity increased as first as the concentration of *p*-TSA was increased up to 0.04 M, but it decreased when the *p*-TSA concentration was further increased up to 0.08 M in the ammonia concentration of 2000 ppm. It is well-known that PPy doped with *p*-TSA which protonated the PPy backbone and endowed it with more positive holes was characterized by a p-type semiconductor. The increase of *p*-TSA resulted in the increase in charge–carrier concentration, which improves the sensitivity of the sensor. When the concentration of *p*-TSA reached the 0.08 M, excessive *p*-TSA molecules existed in the form of free acids. When the excessive free *p*-TSA molecules contacted with the ammonia vapor, it would compete with the –N^+^H– sites in the PPy chain to react with the absorbed ammonia, which would depress the deprotonation process, resulting in a smaller resistance change and a lower sensitivity.

**Figure 6:**
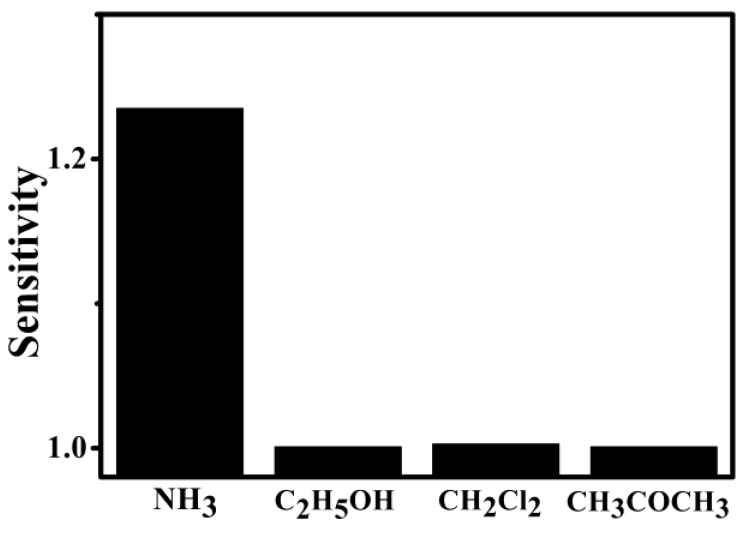
Selectivity of PPy–PAN nanofiber yarn sensor. Concentration of the gas is 500 ppm. (The concentration of *p*-toluenesulfonic acid (*p*-TSA) was 0.004 M).

The selectivity of the PPy–PAN nanoyarn sensor (the concentration of *p*-toluenesulfonic acid (*p*-TSA) was 0.004 M) was operated by testing the sensitivity of the sensor exposed to different kinds of volatile organic compounds with the same concentration in 500 ppm. It is shown in Figure 6 that the response of the nanoyarn sensor towards NH_3_ is obviously higher compared with that towards C_2_H_5_OH, CH_2_Cl_2_, and CH_3_COCH_3_. The difference in the response of the nanoyarn sensor for different gases might result from this characteristic of gas. The adsorption kinetics of gas on the active sensing layer might result in the response for a particular gas or vapor [41]. In our results, a higher sensitivity for NH_3_ might be different adsorption dynamics of the analytes on the surface of the polypyrrole shell layer.

## 3. Discussion

The excellent sensing properties of the PPy–PAN nanofiber yarn to ammonia was the transient response and fast recovery performance, which can be understood by the response mechanism. The response mechanism can be explained from two aspects: the structure of the nanoyarn sensor and the sensing mechanism of the conducting polymer.

The structure of sensing material is core-shell PPy-coated PAN nanofiber yarn. This nanosized coaxial PPy–PAN nanofibers exhibit a higher surface-to-volume ratio, which provide more sites for the adsorption of analyte molecules. The uniaxially aligned nanofibers can facilitate the one-way transmission of electrical signals. The nanoyarn composed of orientated coaxial PPy–PAN nanofibers is orderly fiber assembly, which is beneficial to the diffusion and adsorption of analyte gas on the activated surface of the sensing material.

The sensing mechanism of the conduction polymer can be understood by two possible reversible interaction mechanisms between the PPy and the ammonia, which can be seen as following [19,42]:

(1) $${\text{PPy}}^{+}/{\text{Cl}}^{-}+{\text{NH}}_{3}\left(\delta^{-}\right)⇌{\text{PPy}}^{0}/{\text{NH}}_{3}{\left(\delta^{-}\right)}^{+},{\text{Cl}}^{-}$$

(2) $${\text{PPy}}^{+}/{\text{Cl}}^{-}+{\text{NH}}_{3}\left(\delta^{-}\right)⇌\text{PPy}{\left(-H\right)}^{0}+{{\text{NH}}_{4}}^{+}{\text{Cl}}^{-}$$

(3) $${\text{PPy}}^{+}/{{\text{SO}}_{3}}^{-}+{\text{NH}}_{3}\left(\delta^{-}\right)⇌{\text{PPy}}^{0}/{\text{NH}}_{3}{\left(\delta^{-}\right)}^{+},{{\text{SO}}_{3}}^{-}$$

(4) $${\text{PPy}}^{+}/{{\text{SO}}_{3}}^{-}+{\text{NH}}_{3}\left(\delta^{-}\right)⇌\text{PPy}{\left(-H\right)}^{0}+{{\text{NH}}_{4}}^{+}{{\text{SO}}_{3}}^{-}$$

In Equilibrium Equations (1) and (3), the doublet nitrogen loses its electrons that transfer between the ammonia molecule and PPy’s positive holes [19], which changes the charge–carrier concentration and the resistance of the material. FeCl_3_ was used as an oxidant, and Cl^−^ as the doping anion in PPy. *p*-TSA was used as a doping acid, and ${{\text{SO}}_{3}}^{-}$ as the doping anion in PPy. Polypyrrole is a p-type material, which is characterized by the PPy doped with ${\text{Cl}}^{-}$ and ${{\text{SO}}_{3}}^{-}$, which carries many holes. The conductivity of the doped PPy is susceptible to the reduction gas. When such a sensor material exposed to ammonia (electron-donating molecules), this electron transfer between ammonia molecule and PPy’s positive hole induce a reduction of the sensitive positive charge density, which leads to a decrease in the conductance layer. The positive-charged polymer backbone (PPy^+^) changes into a neutral one (PPy^0^), which reduces charge carriers density (hole) and increases the resistivity by decreasing the electrical conductivity. By purging with air, reversible reaction takes place. The alteration of conductivity of the sensor is attributed to the modulation of the redox levels and partial charge transfer of conducting polymers. The direction of the transfer is determined by the electronegativity and the work function of vapor [43]. Equilibrium Equations (2) and (4) indicate a proton transfer between the polymer and the ammonia so that the ammonium-anion complex forms. In the case of the proton transfer phenomenon, ammonia gas molecules react with protons that are acidic, forming ammonium ions. These reactions result in the formation of a negative charge on the chain of PPy that can integrate with the cavity and undope the PPy. The ammonium-anion complex can freely diffuse on the surface of the PPy. Reverse process takes place on exposure to air and removing the ammonia gas. Ammonium ion decomposes into ammonia gaining protons from the PPy. The proton transfer process is reversible when the ammonium ion only binds very weakly to the negative anion, facilitating the reverse reaction. There is an irreversible change if the material is exposed to a high concentration of ammonia gas or to ammonia and water vapor for a long period of time. The ammonium salt crystal formation will result in the occurrence of an irreversible response. Thus, for long term usability and repeatability, the materials must be fully dried.

## 4. Materials and Methods

### 4.1. Materials

Polyacrylonitrile (PAN, Mw = 75,000 g/mol) was purchased from the Shanghai Chemical Fibers Institute (Shanghai, China). *N*,*N*-dimethylformamide (DMF) was purchased from Shanghai Lingfeng Chemical Reagent Co., Ltd. (Shanghai, China) All reagents, pyrrole, iron(III) chloride (FeCl_3_·6H_2_O), *p*-toluenesulfonic acid (*p*-TSA), dichloromethane, acetone, and ethyl alcohol were purchased from Sinopharm Chemical Reagent Co., Ltd. (Shanghai, China), which were analytically pure.

### 4.2. The Fabrication of the PAN Nanofiber Yarns

First, PAN was dissolved in DMF with a concentration of 10%. The obtained solution was stirred magnetically at room temperature until it became homogeneous. The uniaxially aligned PAN nanofiber yarns were continuously manufactured by the electrospinning nanoyarn machine described in [28]. The distance of two oppositely placed metal needles was 200 mm. The distance between the neutral metal disc (the diameter of 60 mm) and the hollow metal rod (the length of 50 mm, inner diameter of 8 mm, and outer diameter of 12 mm) was 70 mm. Applied voltage at the metal needles was ±10 kV. The solution flow rate was 0.8 mL/h. The rotation speed of the metal disc was 250 r/min. The linear velocity of the take-up roll was 2 m/min.

### 4.3. Polymerization of PPy on the Surface of the PAN Nanofiber Yarn

The coaxial PPy-coated PAN nanofiber yarns were prepared using a simple in situ chemical polymerization. The glass slide (25.4 mm × 76.2 mm × 0.8 mm) winded the PAN nanofiber yarns was dipped into a beaker containing an aqueous solution of pyrrole (0.04 M), FeCl_3_·6H_2_O (0.2 M), a different concentration of *p*-TSA (0.004 M, 0.02 M, 0.04 M, and 0.08 M), which had been magnetically stirred for several minutes in advance. Then, the pyrrole was polymerized on the surface of the nanofiber yarns at 0 °C for 4 h. When the color of the yarns changed from yellowish to black, the samples were removed from the solution and rinsed with deionized water and ethyl alcohol to remove PPy particles and residual reactants and dried in an oven for 2 h.

### 4.4. Characterization

The morphologies and structures of coaxial PPy–PAN nanofiber yarns were observed with environment scanning electron microscopy (ESEM, Quanta-250, FEI, Brno, Czech Republic), field emission scanning electron microscopy (FESEM, S-4800, Hitachi, Ltd., Tokyo, Japan), and transmission electron microscopy (TEM, JEM-2100, JEOL Co., Ltd., Tokyo, Japan). The chemical structures of coaxial PPy–PAN nanofiber yarns were measured using a fourier transform infrared spectroscopy (FTIR, Nicolet 6700, Thermo Fisher Scientific, Inc., Waltham, MA, USA). Gas sensing experiments were operated with CGS-1TP Intelligent Gas Sensing Analysis Equipment (Beijing Elite Tech Co., Ltd., Beijing, China). The sensor sensitivity was defined as S = R_g_/R_a_, where R_g_ and R_a_ were the sensor resistance in ammonia gas and air, respectively. The response time and recovery time of the nanocomposites were defined as the time taken to reach 90% of the total resistance change. The time taken by the sensor to change from R_a_ to $R_{a}+90\%\left(R_{g}-R_{a}\right)\;$ was defined as the response time when the sensor was placed into the ammonia gas. The time taken by the sensor to change from R_g_ to $R_{g}-90\%\left(R_{g}-R_{a}\right)\;$ was defined as the recovery time when the sensor was taken out of the ammonia gas.

## 5. Conclusions

The coaxial PPy–PAN nanofiber yarn was electrospun using a novel nanoyarn machine and in situ chemical synthesis method. PPy was homogeneously and uniformly coated on surface of PAN nanofibers. The highly orientated alignment of PPy-coated PAN nanofibers and one-dimensional structure of nanofiber yarn offered a high surface area for the free access of ammonia to PPy and also opened one-way electron transmission access, which endowed the sensor with excellent sensitivity and fast response/recovery. The prominent characteristics of the PPy–PAN nanoyarn sensor were a feature of transient response. The response time was less than 1 s. In addition, the different concentration of doping acids had an effect on the sensitivity of the nanoyarn sensor. These studies demonstrated that the structure of uniaxially aligned nanofiber yarn and the synergistic effect of nanomaterial and conducting polymers have a great significance in the development of the sensor field.

## Abbreviations

The following abbreviations are used in this manuscript:

PPy	polypyrrole
PAN	polyacrylonitrile
*p*-TSA	*p*-toluenesulfonic acid
PANI	polyaniline
DMF	*N,N-*dimethylformamide
ESEM	environment scanning electron microscopy
FESEM	field emission scanning electron microscopy
TEM	transmission electron microscopy
FTIR	fourier transform infrared spectroscopy
